# Loss of steroid hormone receptors is common in malignant pleural and peritoneal effusions of breast cancer patients treated with endocrine therapy

**DOI:** 10.18632/oncotarget.15548

**Published:** 2017-02-20

**Authors:** Willemijne A.M.E. Schrijver, Karianne Schuurman, Annelot van Rossum, Dutch Distant Breast Cancer Metastases Consortium, Ton Peeters, Natalie Ter Hoeve, Wilbert Zwart, Paul J. van Diest, Cathy B. Moelans

**Affiliations:** ^1^ Department of Pathology, University Medical Center Utrecht, The Netherlands; ^2^ Division of Molecular Pathology, The Netherlands Cancer Institute, The Netherlands

**Keywords:** breast cancer, distant metastases, receptor conversion, effusions

## Abstract

Discordance in estrogen receptor alpha (ERα), progesterone receptor (PR), androgen receptor (AR) and human epidermal growth factor receptor 2 (HER2) status between primary breast cancers and solid distant metastases (“conversion”) has been reported previously. Even though metastatic spread to the peritoneal and pleural cavities occurs frequently and is associated with high mortality, the rate of receptor conversion and the prognostic implications thereof remain elusive.

We therefore determined receptor conversion in 91 effusion metastases (78 pleural, 13 peritoneal effusions) of 69 patients by immunohistochemistry (IHC) and *in situ* hybridization. Data were coupled to clinical variables and treatment history.

ERα, PR and AR receptor status converted from positive in the primary tumor to negative in the effusion metastases or *vice versa* in 25-30%, 30-35% and 46-51% of cases for the 1% and 10% thresholds for positivity, respectively. 19-25% of patients converted clinically relevant from “ER*α*+ or PR+” to ER*α*-/PR- and 3-4% from ER*α*-/PR- to “ERα+ or PR+”. For HER2, conversion was observed in 6% of cases. Importantly, receptor conversion for ERα (*p* = 0.058) and AR (*p* < 0.001) was more often seen in patients adjuvantly treated with endocrine therapy. Analogous to this observation, HER2-loss was more frequent in patients adjuvantly treated with trastuzumab (*p* < 0.001).

Alike solid distant metastases, receptor conversion for ERα, PR, AR and HER2 is a frequent phenomenon in peritoneal and pleural effusion metastases. Adjuvant endocrine and trastuzumab therapy imposes an evolutionary selection pressure on the tumor, leading to receptor loss in effusion metastases. Determination of receptor status in malignant effusion specimens will facilitate endocrine treatment decision-making at this lethal state of the disease, and is hence recommended whenever possible.

## INTRODUCTION

Each year, around 550.000 women die from the consequences of breast cancer [1], largely due to metastatic relapse. In around 30% of patients with metastatic breast cancer, the pleural cavity is involved [2, 3] and less frequently the pericardial and peritoneal cavities [4, 5]. The presence of metastatic breast carcinoma cells in effusions is associated with poor prognosis and a median survival of 5 months [2, 3, 6–8].

Immunohistochemistry (IHC) plays a valuable role in effusion cytology for the identification of metastatic malignancy. Inclusion of hormone receptor status assessment could direct treatment decision-making. This is underlined by the finding that tamoxifen treatment showed a therapeutic benefit in patients with ERα-positive malignant pleural effusions [9–11].

In the clinical management of metastatic breast cancer, the choice of systemic treatment is traditionally based on the tissue characteristics of the primary tumor. Several previous studies have however shown that the expression of predictive tissue markers including ERα, PR and HER2 may differ between the primary breast tumor and solid distant metastases (“receptor conversion”) in a significant proportion of patients [12–14]. Prolonged evolutionary pressure invoked by systemic endocrine therapies may effect hormone receptor expression, and with that, alter drug response. Consequently, alterations of hormone receptor expression in metastatic lesions in relation to the primary tumor may directly result in inappropriate endocrine treatment selection. Several guidelines therefore now recommend to biopsy distant metastases, and to reassess hormone and HER2 receptor status by IHC whenever possible [15, 16].

Androgen receptor (AR) is expressed in 60% of breast cancers and is more frequently expressed in ERα-positive than in ERα-negative tumors. AR signaling pathways show a distinct pattern, depending on the breast cancer subtypes. In ERα-positive breast cancer, AR is thought to antagonize the proliferative effect of ERα and in ERα-negative tumors, AR signaling has a proliferative role [17]. In a comparison of ERα- and AR-positive breast cancer and paired local recurrences or solid distant metastases, AR expression is often maintained even when ERα-expression is lost [18, 19]. This suggests that anti-androgens may be a useful therapeutic strategy for patients with anti-estrogen resistant metastatic disease. Therefore, clinical trials addressing AR-targeted therapies in metastatic breast cancer are currently performed (http://www.cancer.gov/about-cancer/treatment/clinical-trials/, trial IDs NCI-2015-02043 and NCT02605486). However, the role of ERα-inhibitor induced selective pressure on AR receptor status in distant metastases remaines to be elucidated.

To our knowledge, there have been no studies on the influence of adjuvant endocrine therapies on receptor conversion between primary breast tumors and their corresponding malignant effusions so far, while this is a frequent metastatic site [20, 21]. Furthermore, also information about differences between receptor expression in solid and effusion metastases is lacking, due to small sample sizes of the reported studies [22, 23].

Here we report IHC staining of ERα, PR, AR and HER2 complemented with HER2 *in situ* hybridization in 69 patients with primary breast carcinomas and their matched malignant peritoneal and/or pleural effusions and solid distant metastases. We furthermore investigated the influence of adjuvant therapies on receptor conversion. Extensive knowledge of possible receptor conversion in malignant effusions could facilitate optimizing patient tailored therapy strategies for metastatic breast cancer patients.

## RESULTS

### Decreased hormone receptor levels in malignant pleural and peritoneal effusions

In total, 69 female breast cancer patients were included in this study with a median age at diagnosis of the primary tumor of 56 years (Table 1). The primary lesions were predominantly of the ductal type and 38% of patients who underwent sentinel node biopsy had positive lymph nodes. Ninety-one malignant effusions were investigated; 78 of pleural and 13 of peritoneal origin. For sixteen patients, two or more consecutive samples were available.

**Table 1:** Clinical characteristics of the patients and pathological characteristics of primary breast carcinomas in this study

Feature	Grouping	N or value	%
Age at primary diagnosis (in years)	Median Range	56 32-85	
Tumor size (in cm)	Median Range	2.4 0.6-10.0	
Histologic type	Invasive ductal Invasive lobular Invasive ductolobular Not available	52 6 5 6	75 9 7 9
Histologic grade (Bloom & Richardson)	I II III Not available	4 27 30 8	6 39 43 12
Mitotic activity index (per 2mm^2^)	Median Range	10 0-60	
Lymph node status	Negative Positive Not available	17 26 26	24 38 38
Site of distant solid metastases (n=15)	GI-tract/gynaecological Skin Lung Bone Liver	6 9 2 2 2	29 44 9 9 9
Site of metastases in body effusions (n=69)	Pleural effusion Ascites	78 13	86 14
Time between diagnosis of primary and first effusion metastasis (in months)	Median Range	45 0-241	
Survival time between diagnosis of first effusion metastasis and end of follow-up (in days)	Median Range	120 0-4477	
Adjuvant endocrine therapy	Yes No Unknown	33 18 18	48 26 26
Adjuvant chemotherapy	Yes No Unknown	31 18 20	45 26 29
Adjuvant targeted therapy	Yes No Unknown	8 40 21	12 58 30

**Figure 1:**
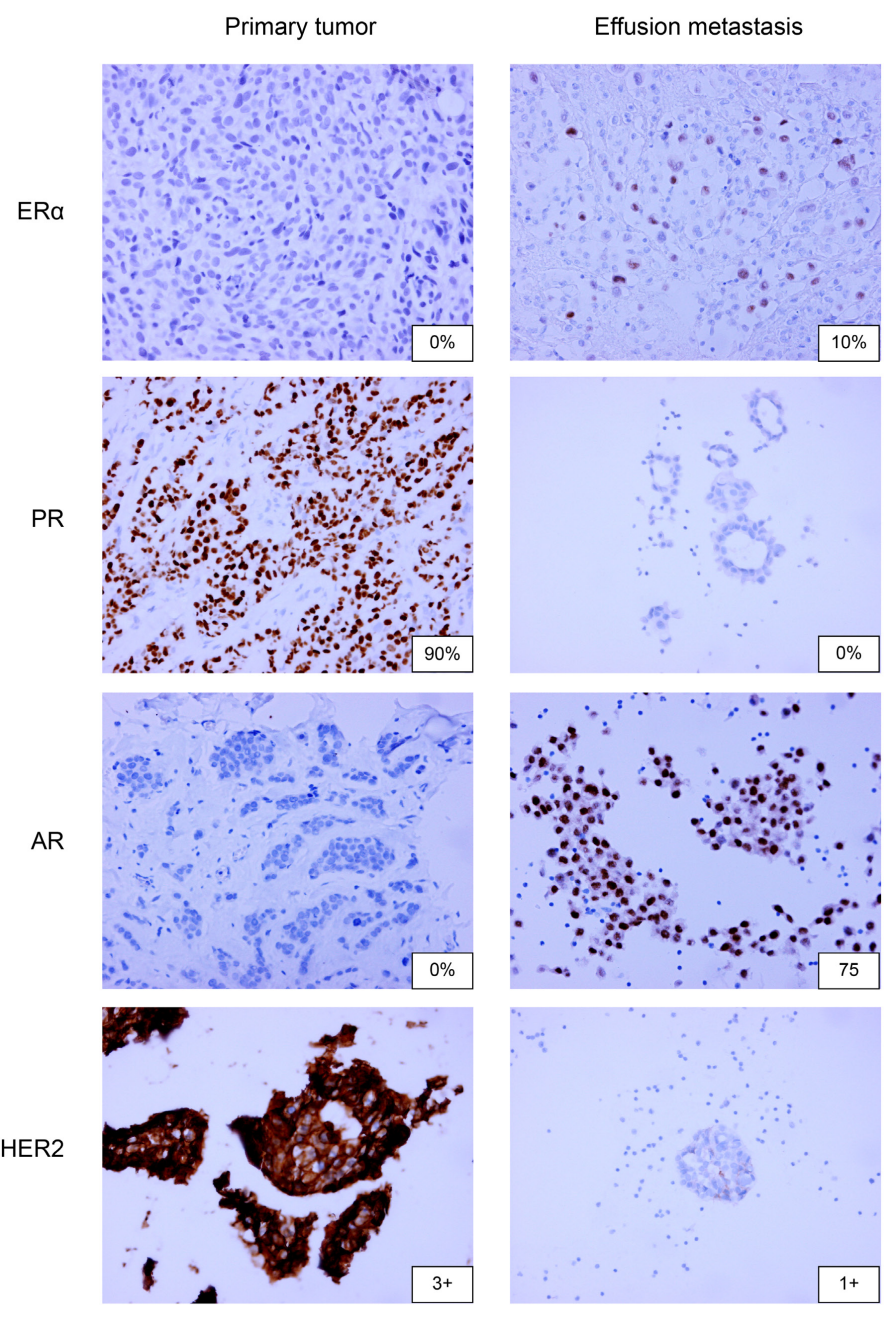
ERα, PR, AR and HER2 immunohistochemistry on paired primary breast tumors and pleural or peritoneal metastases 20x magnification is used.

ERα positivity was observed in the vast majority of primary tumors (65% or 71% for the 10% or 1% thresholds for positivity, respectively). Solid metastases were more often ERα negative (*p* = 0.022 for the 10% threshold and *p* = 0.079 for the 1% threshold; ERα positivity of 38% for both thresholds), as was the case for effusion metastases (*p* = 0.024 and *p* = 0.097; ERα positivity of 46% or 57% for the 10% or 1% thresholds for positivity, respectively). PR positivity was generally lower than ERα in the primary tumors (*p* = 0.004 and *p* = 0.154; PR positivity of 39% or 58%, respectively). AR was expressed in 60% or 71% of primary tumors and 10% or 26% of effusion metastases (Figure 1; *p* < 0.001 for the 10% and 1% thresholds for positivity, respectively).

### Frequent hormone receptor discordance in paired breast cancer and effusion metastases

ERα, PR and AR showed a significantly lower expression in effusion samples compared to the paired primary tumors (*p* < 0.001 for all three receptors; n = 69). For the 10% theshold for positivity, 30% (21/69) of patients showed ERα conversion, with 26% (18/69) from positive in the primary tumor to negative in the effusions and 4% (3/69) from negative to positive. For PR, conversion rates were similar (total 30%; 21/69), with 25% (17/69) of samples converting from positive to negative and 5% (4/69) from negative to positive. For AR, discordance was even higher with 50% (35/69) of samples converting from positive to negative, and 1% (1/69) conversion from negative to positive. When comparing solid metastases to paired effusion metastases, ERα, PR and AR protein expression diverged in 20% of cases (3/15; Table 2).

**Table 2:** Immunohistochemical hormone receptor status (ERα, PR and AR) of primary tumors and solid and effusion metastases of patients analyzed in this study

10%			Primary *N*= 69			Solid metastasis *N*= 15			1%			Primary*N*= 69			Solid metastasis *N*= 15		
			-	+	*p*	-	+	*p*				-	+	*p*	-	+	*p*
ERα	Solid metastasis N=15	-	7	2	0.50				ERα	Solid metastasis *N* = 15	-	6	3	0.25			
		+	0	6							+	0	6				
	Effusion *N* = 69	-	21	18	0.001	8	2	1		Effusion *N* = 69	-	17	14	0.013	7	1	1
		+	3	27		1	4				+	3	35		2	5	
PR	Solid metastasis *N* = 15	-	9	2	0.50				PR	Solid metastasis *N* = 15	-	6	2	1			
		+	0	4							+	1	6				
	Effusion *N* = 69	-	38	17	0.007	10	2	1		Effusion *N* = 69	-	26	21	<0.001	8	2	0.50
		+	4	10		1	2				+	3	19		0	5	
AR	Solid metastasis *N* = 12	-	7	1	1				AR	Solid metastasis *N* = 12	-	6	2	1			
		+	2	2							+	1	3				
	Effusion *N* = 64	-	25	31	<0.001	8	2	0.50		Effusion *N* = 64	-	17	29	<0.001	7	1	1
		+	1	7		0	1				+	2	16		1	2	

Data are shown for 10% and 1% cut-offs of positivity.

For the 1% threshold for positivity, less conversion was seen. 25% (17/69) of patients showed ERα conversion, with 21% (14/69) from positive in the primary tumor to negative in the effusions and 4% (3/69) from negative to positive. For PR, 35% (24/69) converted in total, with 30% (21/69) of samples converting from positive to negative and 5% (3/69) from negative to positive. For AR, 43% (30/69) of samples converted from positive to negative, and 3% (2/69) from negative to positive. When comparing solid metastases to paired effusion metastases, ERα diverged in 20% (3/15), PR in 13% (2/15) and AR in 20% (3/15) of cases (Table 2).

Clinically relevant conversion (from “ERα+ or PR+” to ERα-/PR-, or from ERα-/PR- to “ERα+ or PR+”) was perceived in 28% (19/69) of patients for the 10% threshold and in 23% (16/69) of patients for the 1% threshold. 25% (17/69) and 19% (13/69) of patients, respectively, converted from “ERα+ or PR+” to ERα-/PR- and 3% (2/69) and 4% (3/69) of patients from ERα-/PR- to “ERα+ or PR+”.

HER2 discordance was seen in 6% (4/69) of cases, were 3% (2/69) shifted from positive to negative and 3% (2/69) from negative to positive (Table 3). Concordance between IHC and FISH for 0, 1+ (being non-amplified) and 3+ cases (being amplified) was high (88%, 23/26, Table 4).

**Table 3:** HER2 receptor status of primary tumors and solid and effusion metastases of patients analyzed in this study

IHC			Primary *N*= 69			Solid metastasis *N*= 15			FISH			Primary *N*= 66			Solid metastasis *N*= 15		
			-	+	*p*	-	+	*p*				-	+	*p*	-	+	*p*
HER2	Solid metastasis *N* = 15	-	12	0	1				HER2	Solid metastasis N=15	-	13	0	1			
		+	1	2							+	0	2				
	Effusion *N* = 69	-	53	3	0.73	11	1	1		Effusion N=66	-	56	2	1	13	0	1
		+	5	8		1	2				+	2	6		0	2	

Data are shown for immunohistochemistry and FISH.

**Table 4:** Differences in HER2 immunohistochemistry and FISH for 2+/3+ or discordant cases

Primary tumor		Solid metastasis		Effusion metastasis	
IHC	FISH	IHC	FISH	IHC	FISH
3+	amp			3+	amp
3+	amp			3+	amp
3+	amp	3+	amp	3+	amp
1+	no amp			2+	amp
0		1+		2+	no amp
3+	amp			3+	no amp
3+	amp	3+	amp	2+	amp
3+	amp			3+	amp
3+	amp			3+	amp
0		2+	no amp	0	
1+	no amp			2+	no amp
2+	no amp			0	
3+	amp			2+	no amp
3+	no amp			3+	amp

**Figure 2:**
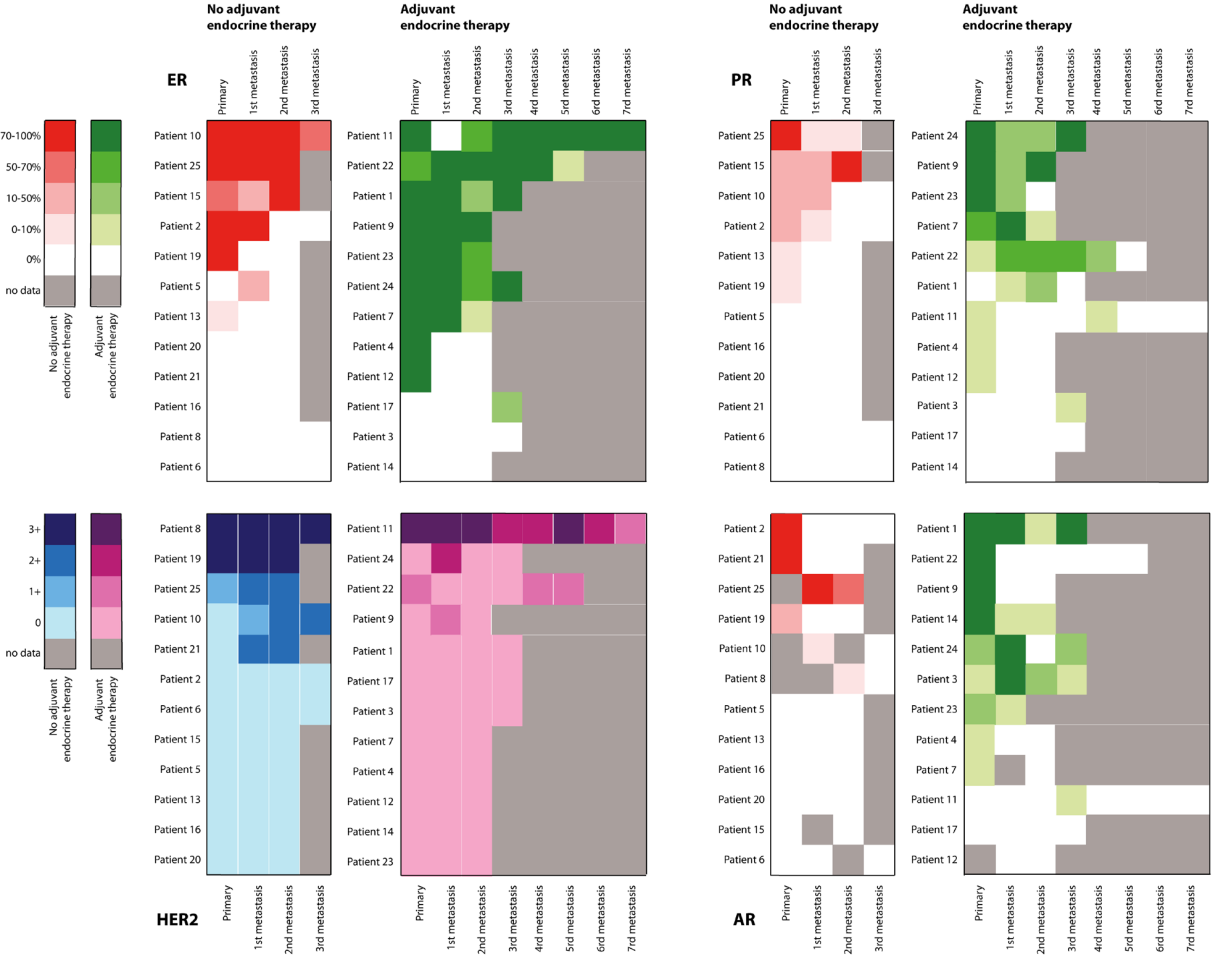
Heatmap of expression of ERα, PR, AR and HER2 in paired primary breast tumors and multiple pleural or peritoneal metastases per patient: progression over time For ERα, PR and AR, green represents patients adjuvantly treated with endocrine therapy; red represents patients not adjuvantly treated with endocrine therapy. For HER2, purple represents patients adjuvantly treated with endocrine therapy; blue represents patients not adjuvantly treated with endocrine therapy.

Out of 69 patients in our cohort, 27 patients had material available of more than one metastasis (multiple solid or effusion metastases; Supplementary table S1). Figure 2 depicts ERα, PR and AR staining percentages and HER2 DAKO-scores for these patients. Large variation occurred during tumor progression from primary tumor to solid and effusion metastases, with no clear trend over time.

### Adjuvant endocrine therapy is associated with receptor conversion in pleural metastases

Adjuvant endocrine therapy was given to 66% (33/50) of the patients (of whom treatment history was known), while 63% (31/49) received adjuvant chemotherapy. For 45% (5/11) of HER2 amplified cases, trastuzumab was prescribed. In Supplementary Table S2 we provide the adjuvant therapy administration per receptor status for both the 1% and 10% thresholds for positivity.

**Figure 3:**
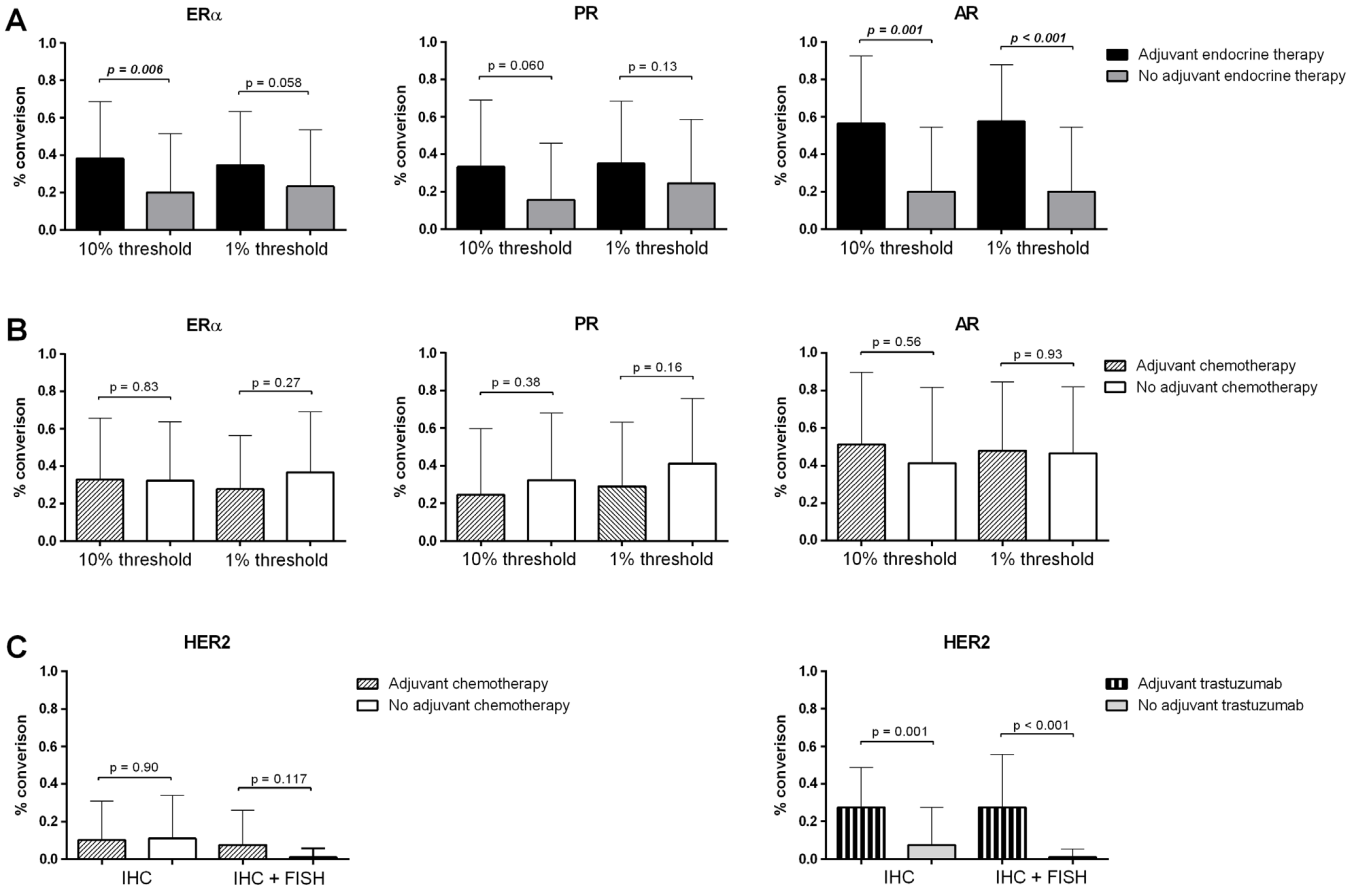
Conversion percentages for ERα, PR, AR and HER2 compared to adjuvant therapy history a. Conversion percentages for ERα, PR and AR of patients that did and did not receive adjuvant endocrine therapy. Data for the 1% and 10% thresholds for positivity are shown. b. Conversion percentages for ERα, PR and AR of patients that did and did not receive adjuvant chemotherapy. Data for the 1% and 10% thresholds for positivity are shown. c. Conversion percentages for HER2 of patients that did and did not receive adjuvant chemo- or trastuzumab therapy. Data for the for IHC only and IHC in combination with FISH are shown.

Patients adjuvantly treated with endocrine therapy showed more often conversion of ERα (*p* = 0.006 or p = 0.058 for the 10% or 1% thresholds for positivity, respectively) and AR (*p* = 0.001 or *p* < 0.001), but not of PR (*p* = 0.060 or *p* = 0.130; Figure 3). Adjuvant chemotherapy did not show such association (ERα: *p* = 0.835 or *p* = 0.271, PR: *p* = 0.383 or *p* = 0.156 and AR: *p* = 0.557 or *p* = 0.927 for the 10% or 1% thresholds for positivity, respectively).

For HER2, adjuvant trastuzumab treatment also influenced the change of receptor status (*p* < 0.001). Again, this effect was not seen for chemotherapy (*p* = 0.117).

## DISCUSSION

Luminal breast cancer is hallmarked by expression and growth dependency on ERα, which represents one of the cornerstones of adjuvant therapy in the treatment of breast cancer. Now that guidelines allow for the use of at least 5 years of endocrine therapeutics [24], and even 10 years for a subset of patients [25], it is not unlikely that such continuous and longitudinal ERα-inhibition would directly invoke a strong evolutionary pressure on the tumor. For approximately 30% of patients, metastatic relapse of the tumor is observed [26], which also implies that the tumor cells managed to survive and proliferate despite multiple years of ERα inhibition. Since the efficacy of adjuvant endocrine therapy was only established during the end of the last century, leading to an increase in prescription [27], studies including samples before that time could not confirm the evolutionary pressure of these therapies.

Next to ERα, also PR, AR and HER2 are treatment targets in the battle against breast cancer. For second or higher lines of therapy, megestrol acetate [28], bicalutamide [29] and trastuzumab [30] are only a few examples of drugs that have demonstrated their clinical utility in breast cancer treatment and continuous research is being performed to develop and optimize new therapies. Furthermore, PR and AR also have the potential to predict response to ERα-targeted therapy; high PR expression in the presence [31] or even absence of ERα [32] is thought to predict an increased probability of benefit from anti-estrogen, while AR protein expression can induce tamoxifen resistance [33].

Receptor conversion in solid distant metastases is now a well-known phenomenon and may lead to suboptimal treatment, and most guidelines now recommend to biopsy distant metastases at presentation of metastatic disease [16, 34, 35]. Receptor status in malignant effusion specimens used to be determined only rarely, as in most cases characteristics of the primary tumor were deemed sufficient. However, in this study we demonstrated that receptor conversion in effusions is also a frequent phenomenon. Especially the high AR discordance we found is new and very relevant, since AR-targeted therapies are recently gaining interest for the treatment of ERα-negative and endocrine therapy resistant breast cancer [36]. Even more interesting, in contrast to the high AR discordance in effusion metastases, relatively stable AR expression was described in solid metastases [18, 19], while ERα, PR and HER2 conversion showed roughly the same pattern in effusion and solid metastases [12–14].

We show for the first time that receptor conversion for ERα and AR in malignant effusions was more often seen in patients adjuvantly treated with endocrine therapy and for HER2 in patients treated with trastuzumab. For ERα, PR and HER2 this was previously shown in primary breast cancer versus solid metastases [37–43]. Conversion occurred most often from positive to negative and could be explained by outgrowth of metastatic negative clones from the primary tumor under the selection pressure of prior therapies [44, 45]. However, ERα-inhibitor induced AR conversion was not shown before and the mechanism of this finding remains elusive. Videlicet, tamoxifen and aromatase-inhibitors are not known to affect AR activity and are therefore not thought to inflict evident evolutionary selection pressure. Another explanation for conversion could be clonal dedifferentiation and selection of or evolution to more aggressive phenotypes [44, 46–48]. Also inadequate sampling of a heterogeneous tumor potentially leads to differences in receptor expression, which may explain some but clearly not all of the differences between primary breast cancer and metastases. Only one previous study reported ERα and PR receptor expression between primary breast tumors and 31 pleural effusion metastases, without mentioning treatment history. With expression rates of 59% and 51% for ERα and PR respectively and receptor conversion of 35% and 42%, these findings corresponded to our results mostly in relation to ERα [49].

In patients with multiple effusion samples and solid metastases available, large variation in hormone and HER2 receptor expression occurred during tumor progression from primary tumor to solid and pleural metastases, with no clear trend over time. This could be explained by the different locations of metastases, since it was shown before that tumors with specific hormone receptor expression patterns show a distinct dissemination pattern [44]. Furthermore, most patients received multiple lines of therapy, potentially all imposing divergent evolutionary selection pressures on the metastatic cells. Also, in our cohort not all patients with ERα- and/or HER2-positive primary breast cancer received adjuvant endocrine or HER2-targeted therapy. This can be explained by the long sample inclusion period (1989-2016). Furthermore, we did not have access to treatment information of all included patients. Future studies addressing types, duration and number of therapies of all included patients would yield priceless information about the influence of systemic drugs on tumor progression.

Since generally less steroid receptor positivity was seen in peritoneal and pleural effusion metastases compared to their matched primary breast carcinomas, the question arises whether IHC staining on histologically processed cytology specimens is reliable. However, several studies have compared ERα, PR and HER2 status in cell blocks to tissue blocks and found high concordance rates between cytology and histology specimens [50, 51]. To prevent potential differences caused by such technical issues, we performed IHC staining on freshly cut cell block sections, used internal and external controls, assessed only the invasive (not the *in situ*) component and performed ISH on samples that scored 2+ or 3+ [52]. Since heterogeneous expression is not uncommon [53], we included only samples containing at least 20 tumor cells.

In summary, we have shown for the first time that ERα, PR, AR and HER2 expression in primary breast cancers is frequently lost in peritoneal and pleural effusion metastases. For ERα, PR and HER2 this is in line with previous findings in solid distant metastases, but AR conversion in late stages of tumor progression is a new observation. We demonstrate that this loss may be inflicted by the evolutionary selection pressure of adjuvant endocrine or targeted therapies, as such accounting for acquired therapy resistance. Considering Enzalutamide treatment, which blocks AR nuclear import, inhibits estrogen-driven MCF-7 cell proliferation (ref:https://breast-cancer-research.biomedcentral.com/articles/10.1186/bcr3599), it is conceivable that AR-loss would have a comparable effect on abrogating ERα function. Since more than 35% of hormone receptor positive primary tumors convert to ERα and/or PR negative metastases or vice versa, determination of receptor status in malignant effusion specimens may help to optimize patient tailored hormonal treatment and is therefore recommended whenever possible. Especially the new finding of treatment-induced loss of AR protein expression as shown here, might have ramifications for clinical studies addressing AR-targeted therapies in metastatic breast cancer.

## MATERIALS AND METHODS

### Material

In total, 91 malignant effusion specimens derived from 69 female breast cancer patients were used for this study. Retrospectively, 71 cell blocks of pleural and peritoneal effusions from 56 patients were obtained from the departments of Pathology of the University Medical Center Utrecht (39 patients), Rijnstate Hospital Arnhem (1 patient), Radboud University Medical Center Nijmegen (2 patients), Bronovo Hospital The Hague (1 patient), Meander Medical Center Amersfoort (2 patients), OLVG Amsterdam (1 patient), Pathology Laboratory Friesland (2 patients), Groene Hart Hospital Gouda (1 patient), Leiden University Medical Center (1 patient), St. Franciscus Hospital Rotterdam (5 patients), Isala Clinics Zwolle (1 patient), all in The Netherlands. Fourteen effusion samples from nine patients were collected prospectively in the Netherlands Cancer Institute in Amsterdam and six samples from four patients from the University Medical Center Utrecht. Selection criteria were: availability of tissue from both primary tumor and effusion metastases, more than 20 tumor cells per cell block, enough tissue to cut sections for IHC analyses on ERα, PR, AR, HER2 and potentially FISH. Original diagnoses had been made between December 1989 and February 2016.

Cytology samples were initially fixed in isopropanolol and embedded in paraffin by Cellient, (Hologic). For each case, hematoxylin-eosin stained slides of the paraffin blocks were reviewed by a single experienced pathologist (PvD) to confirm the presence of malignancy in all cytology samples. Only samples containing at least 20 tumor cells were selected. Ber-EP4 monoclonal antibody staining (Monosan), labelling epithelial tissues without reacting with mesothelial cells, was used to confirm presence of tumor cells.

All samples were compared with the corresponding primary tumor and, when present, with one or more paired solid distant metastases (fifteen patients). All samples were recut and restained, using current standardized techniques (see below).

This study was performed in accordance with the medical ethical guidelines of the University Medical Center Utrecht. The use of anonymous or coded left over material for scientific purposes is part of the standard treatment agreement with patients and therefore ethical approval was not required [54].

### Immunohistochemistry

IHC for ERα, PR, HER2, AR and Ber-EP4 was carried out on full 4-μm sections with the Ventana (Ventana Medical Systems) according to the manufacturer's instructions with mouse monoclonal antibodies against Ber-EP4/Ep-CAM (1:800, BS14, Monosan) and AR (1:20, AR27, Novocastra) and rabbit monoclonal antibodies against ERα (ready-to-use, SP1, Roche), PR (ready-to-use, 1E2, Roche) and HER2 (1:50, SP3, ThermoFisher). Appropriate controls were used throughout.

### Scoring

Scoring of IHC slides was performed by consensus of two observers (PvD & WS) in random order, blinded to other data. The adequacy of staining in the primary carcinoma was checked by also evaluating the normal breast parenchyma when present.

For ERα, PR and AR, the percentage of positively stained nuclei was estimated side by side with the BER-Ep4 stained slide as a reference. Samples with 10% or more immunopositive malignant cells, regardless of staining intensity, were classified as ERα or PR positive (European standard). The same was done for the 1% USA threshold.

HER2 expression was scored using the DAKO scoring system as 0, 1+, 2+ and 3+ [55]. HER2 expression was considered negative when 0 or 1+, equivocal when 2+ and positive when 3+. We regarded HER2 conversion as a shift from 0/1+/2+ without amplification by FISH to 2+ with amplification/3+ or *vice versa*.

### *In situ* hybridization

All cases with 2+/3+ and discordant results in primary tumors compared to paired metastases were subjected to fluorescence *in situ* hybridization using a HER2/CEP17 dual FISH probe (Cytocell) on 4-μm slides. Analysis was performed on a Leica DM5500 B microscope system with Application Suite Advanced Fluorescence Software (Leica Microsystems).

In short, formalin-fixed paraffin-embedded slides were deparaffinized and pretreated with citrate and protease buffers. Next, they were dehydrated and hybridized with 10μl probe in a ThermoBrite (Abbott Laboratories) at 37°C overnight. The next day, slides were washed in saline-sodium citrate buffers, counterstained with DAPI, dehydrated and mounted with Vectashield Mounting Medium (Vector Laboratories). One hundred tumor cell nuclei per tumor were assessed for HER2 gene and CEP17 probe signals at 100x magnification. The HER2/CEP17 ratio was calculated as well. A ratio below 1.8 was defined as a normal copy number, a ratio of 1.8–2.2 as an equivocal copy number and a ratio above 2.2 as gene amplification, according to the ASCO & CAP guidelines [56].

### Statistics

Expression frequency of ERα, PR, AR and HER2 was compared in the primary tumors versus paired effusion and solid metastases using Wilcoxon signed-rank test. Comparison of IHC expression in peritoneal and pleural effusions was performed using Mann-Whitney U test. Dichotomized conversion data (from positive to negative and vice versa) were calculated for 1% and 10% thresholds for positivity and compared by Mc Nemar's test. As steroid receptor conversion is clinically important if a patient converts from “ERα+ or PR+” to ERα-/PR-, or from ERα-/PR- to “ERα+ or PR+”, we calculated the frequency for these conversions as well. Statistical analysis was performed using IBM SPSS Statistics version 21 and visualized using GraphPad Prism 6.

We thank Stichting PALGA for the national query for cases.

## SUPPLEMENTARY MATERIALS TABLES


